# The conditional growth suppressor *E7.25d.7* is an allele of the sterile-20 kinase *misshapen*

**DOI:** 10.17912/micropub.biology.000424

**Published:** 2021-07-22

**Authors:** Evan Kiely, Melissa Gilbert-Ross

**Affiliations:** 1 Department of Hematology and Medical Oncology, Emory University School of Medicine; 2 Present address: Winship Data and Technology Applications Shared Resource, Winship Cancer Institute of Emory University

## Abstract

The notion of a two-hit or multi-hit model of carcinogenesis dates to at least the 1970’s and work done by Alfred Knudson. This concept was considered in the design and execution of a previous FLP/FRT screen in *Drosophila melanogaster* for conditional growth suppressors. During the course of this work, the lethal allele *E7.25D.7* was identified as being of phenotypic interest. Here we report the genetic mapping of *E7.25D.7*, an allele of the sterile-20 kinase *misshapen *(*msn*).

**Figure 1. E7.25d.7 synergizes with a block in cell death to override organ size control and maps to the msn locus:**
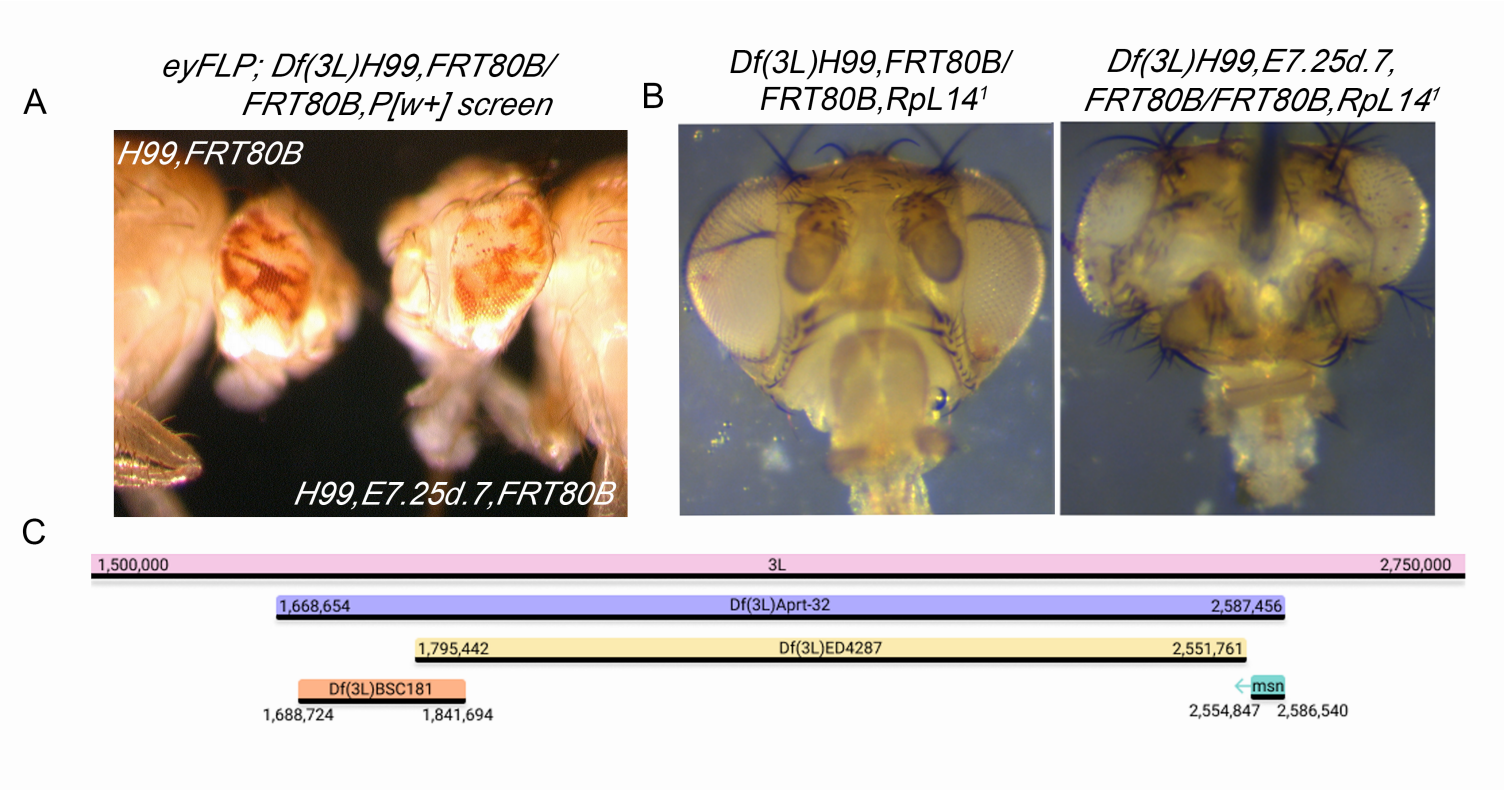
(A) Representative brightfield image of mosaic *Df(3L)H99,FRT80B* and *Df(3L)H99,E7.25d.7,FRT80B* eyes from the original w>r screen (B) Representative brightfield images of eyes/head capsule from *Df(3L)H99,FRT80B/RpL14^1 ^*and *Df(3L)H99,E7.25d.7/RpL14^1^* flies Surviving *RpL14^1^* tissue is marked by *w^+mC^* and all images were captured at 4X magnification (C) Genomic interval and deficiencies used to finely map *E7.25d.7* to the *msn* locus. These intervals, as illustrated, are uniformly scaled, and their locations are accurate both relative to one another and 3L. This was achieved using a standard normalization, with arbitrary minimum and maximum values (shown on 3L) chosen to represent the approximate neighborhood of the examined intervals.

## Description

The lethal allele *E7.25d.7* was isolated in a previously described FLP/FRT screen for conditional growth suppressors on chromosome 3L (Gilbert *et al.*, 2011). Briefly, male *w^1118^*;; *Df(3L)H99,FRT80B/TM6B* flies were fed 25mM EMS and mated en mass to *eyFLP;;ubi-GFP, FRT80B* virgin females to screen a total of ~20,000 chromosome arms. Although most alleles from the screen were selected based on overgrowth of mutant white tissue relative to red wild-type twin spots, *E7.25d.7* retained a mostly 50:50 white:red ratio, and apparent cell shape and bristle defects (Fig. 1A). To test whether our *E725d.7* allele could synergize with *Df(3L)H99* to override organ size controls, we crossed *H99,E7.25d.7,FRT80B/TM6B* to *eyFLP;; RpL14^1^,FRT80B/TM6B*, a Minute allele (Saeboe-Larssen *et al.*, 1997), thus allowing us to create entire heads that were mutant for *E725d.7* and lacked developmental cell death. Compared to *Df(3L)H99,FRT80B/FRT80B,RpL14^1^* control heads, *H99,E7.25d.7,FRT80B*/FRT80B,*RpL14^1^* heads were larger and malformed, contained increased numbers of thick bristles, evidence of necrosis, and tumor-like morphological abnormalities (Fig. 1B). These phenotypes had different levels of expressivity, with females consistently exhibiting the most severe phenotypes.

In order to identify the gene, *Df(3L)H99* was recombined off of the mutant chromosome, which was then checked for maintenance of lethality. *E7.25d.7,FRT80B/TM6B* female flies were then crossed in single vials to males from each of the 77 stocks of the Bloomington 3L Deficiency Kit (Cook *et al.*, 2012). *E7.25d.7* failed to complement *Df(3L)Aprt-32* (3L: 1,668,654 … 2,587,456; Cook, 2016), but complemented *Df(3L)BSC181* (3L: 1,688,724 … 1,841,694; BDSC, 2008) and *Df(3L)ED4287*(3L: 1,795,442 … 2,551,761; DrosDel Project, 2007) (Table 1). We obtained loss-of-function alleles for two candidates, *daughter of sevenless* (*dos*^P115^) and *mishappen* (*msn*^102^). *msn*^102^failed to complement *E7.25d.7*. In a separate experiment *E7.25d.7* failed to complement an independent allele of *msn* (*msn*^172^) (Table 2).

**Table:** 

**Selected 3L deficiency stocks**			
**Deficiency**	**BDSC Stock #**	**Genomic region**	**Complementation Test with E7.25d.7**
Df(3L)BSC181	9693	3L: 1,688,724 … 1,841,694	Complement
Df(3L)Aprt-32	5411	3L: 1,668,654 … 2,587,456	Non-Complement
Df(3L)ED4287	8096	3L: 1,795,442 … 2,551,761	Complement
**Single genes tested within the Df(3L)Aprt-32 deficiency region**			
Gene	BDSC Stock #	Allele	**Complementation Test with E7.25D.7**
dos	6845	dos^P115^	Complement
msn	5945	msn^102^	Non-Complement
msn	5947	msn^172^	Non-Complement

*msn* encodes a ste-20-like Ser/Thr kinase of the MAPK4 family, and alleles were originally identified in two independent screens for genes required for eye development (Xu and Rubin, 1993), and those regulated by the photoreceptor transcription factor Glass (Treisman *et al.*, 1997). The phenotypic abnormalities in the eye of the original *msn* alleles included cell shape and orientation changes in photoreceptors. It was also reported that *msn* transcripts are expressed in cells undergoing cell migration and shape changes, and ultimately it was hypothesized that Msn plays a role in cell signaling during cytoskeletal rearrangement. Msn has recently shown to act, along with other MAPK4-family kinases, to phosphorylate the Hippo pathway kinase Warts (Wts) to restrict Yorkie (Yki) activity (Meng *et al.*, 2015; Li *et al.*, 2015). Importantly, Msn activity is partially redundant to both Wts and other MAPK4 family members in restricting Yki activity, which could explain the conditional nature of the allele.

## Reagents

*Df(3L)H99,FRT80B/TM6B (Gilbert *et al.*, 2011)*

*E7.25D.7,FRT80B/TM6B-GFP* (this manuscript)

*Df(3L)H99,E7.25D.7,FRT80B/TM6B, Tb* (this manuscript)

*yweyFLP;; P[m-w+]RpL14^1^FRT80B, /TM6B* (gift from K. Moberg)

*eyFLP;;P[m-w+;ubiGFP]FRT80B* (gift from K. Moberg)

Bloomington Drosophila Stock Center 3L Deficiency Kit

w*; P{lacW}dos^P115^ Diap1^1^. Cu^1^ sr^1^/TM6B, Tb^1^(BDSC #6845)

w*;msn^102^P{neoFRT}80B/TM6B (BDSC #5945)

w*;*msn^172^*,*P{neoFRT}80B/TM6B*, *Tb^1 ^*(BDSC #5947)
